# miRNA expression profiles in cerebrospinal fluid and blood of patients with Alzheimer’s disease and other types of dementia – an exploratory study

**DOI:** 10.1186/s40035-016-0053-5

**Published:** 2016-03-15

**Authors:** Sofie Sølvsten Sørensen, Ann-Britt Nygaard, Thomas Christensen

**Affiliations:** Department of Neurology, Copenhagen University Hospital, Nordsjællands Hospital, Dyrehavevej 29, 3400 Hillerød, Denmark; Department of Clinical Biochemistry, Copenhagen University Hospital, Nordsjællands Hospital, Dyrehavevej 29, 3400 Hillerød, Denmark

**Keywords:** Alzheimer’s disease, Dementia, Neurodegenerative disease, miRNA expression, Diagnostic biomarker, Cerebrospinal fluid, Blood

## Abstract

**Background:**

MicroRNAs (miRNAs) are small non-coding RNA molecules that function as posttranscriptional regulators of gene expression. Measurements of miRNAs in cerebrospinal fluid (CSF) and blood have just started gaining attention as a novel diagnostic tool for various neurological conditions. The purpose of this exploratory investigation was to analyze the expression of miRNAs in CSF and blood of patients with Alzheimer’s disease (AD) and other neurodegenerative disorders in order to identify potential miRNA biomarker candidates able to separate AD from other types of dementia.

**Methods:**

CSF was collected by lumbar puncture performed on 10 patients diagnosed with AD and 10 patients diagnosed with either vascular dementia, frontotemporal dementia or dementia with Lewy bodies. Blood samples were taken immediately after. Total RNA was extracted from cell free fractions of CSF and plasma, and a screening for 372 known miRNA sequences was carried out by real time quantitative polymerase chain reactions (miRCURY LNA™ Universal RT miRNA PCR, Polyadenylation and cDNA synthesis kit, Exiqon).

**Results:**

Fifty-two miRNAs were detected in CSF in at least nine out of ten patients in both groups. Among these, two miRNAs (let-7i-5p and miR-15a-5p) were found significantly up-regulated and one miRNA (miR-29c-3p) was found significantly down-regulated in patients with AD compared to controls. One hundred and sixty-eight miRNAs were frequently detected in the blood, among which miR-590-5p and miR-142-5p were significantly up-regulated and miR-194-5p was significantly down-regulated in AD patients compared to controls.

**Conclusions:**

Detection of miRNA expression profiles in blood and in particular CSF of patients diagnosed with different types of dementia is feasible and it seems that several expressional differences between AD and other dementia types do exist when measured in a clinically relevant setup. In this explorative pilot study, the deregulated miRNAs in CSF of AD patients may be associated with relevant target genes related to AD pathology, including APP and BACE1, which suggests that miRNAs are interesting candidates for AD biomarkers in the future.

**Electronic supplementary material:**

The online version of this article (doi:10.1186/s40035-016-0053-5) contains supplementary material, which is available to authorized users.

## Background

Alzheimer’s disease (AD) is an age-related progressive neurodegenerative disorder characterized by cognitive impairment and neuropsychiatric symptoms leading to restrictions in the activities of daily living. AD is the most common type of dementia accounting for 50–70 % of all dementia cases. Approximately 10 % of elderly people (65+ years) are affected by AD with an age-specific prevalence that almost doubles every five years after 65 [1]. Because of the ageing population worldwide, the increasing incidence and socioeconomic impact of AD and other dementias represents a growing challenge to public health all over the world [2, 3].

The complex neurobiology of AD, which is not fully understood, includes the development of extracellular amyloid plaques and intracellular neurofibrillary tangles, vascular changes, neuronal inflammation, neurochemical changes, and progressive brain atrophy [4–7].

The prospect of getting an effective treatment is not only complicated by the lack of knowledge about the underlying pathophysiological mechanisms of AD but also by the difficulty of accurately diagnosing AD at an early stage.

When compared to healthy individuals, existing cerebrospinal fluid (CSF) biomarkers support the clinical diagnosis of AD with a high predictive accuracy, however, in differentiating AD from other types of dementia (e.g. vascular dementia, frontotemporal dementia and dementia with Lewy bodies) the current biomarkers have their limitations. In a newly published meta-analysis the sensitivity and specificity of CSF amyloid beta (Aβ1-42) for separating AD from other dementias were estimated to be 73 and 67 %, respectively [8]. For total CSF tau the sensitivity has been estimated to be 70–75 % and the specificity 74–90 % and for phospo-tau 79–88 % and 78–83 %, respectively, for separating AD from other dementias [9].

Development of new and better biomarkers for AD and other dementias would result in more accurate diagnoses facilitating the possibility for an early and specialized treatment effort for the growing number of patients with dementia. Especially now that medical treatment options for AD are available, it is particularly important to identify these patients at an early stage.

MicroRNAs (miRNAs) are small non-coding RNA molecules of approximately 22 nucleotides in length that function as posttranscriptional regulators of gene expression. In mammalian cells, miRNAs work through base pairing with complementary sequences within messenger RNA (mRNA) molecules, usually resulting in gene silencing via translational repression. Recent studies indicate that the expression patterns of miRNAs change in relation to various neurological diseases including Alzheimer’s disease. Altered expression of specific miRNAs in the brain of AD patients compared to controls has been reported, including down-regulation of miR-15a/b, mR-16, miR-29a/b, miR-195, miR-103 and miR-107, which have all been shown to target the β-site amyloid precursor protein cleaving enzyme 1 (BACE1) involved in the formation of amyloid plaques [10–15]. Lukiw et al. [16, 17] studied the miRNA expression in hippocampal tissue of AD patients, and found up-regulation of specific pro-inflammatory miRNAs including miR-9, miR-125b, miR-146a, and miR-155, which all seem to be induced by NF-κB, thus indicating a possible role of these miRNAs in neuronal inflammation in AD. On the other hand, Cogswell et al. [18] have found a significant down-regulation of miR-9 in the human hippocampus of AD patients. miR-9 is known from several experimental studies to regulate neuronal differentiation [19].

Measurements of miRNAs in biofluids such as CSF and blood have just started to gain attention as a novel diagnostic tool. Since CSF is separated from blood circulation by the blood–brain barrier (BBB), it makes good biological sense that the CSF could contain unique signatures of miRNA expression specific for various CNS pathologies. Therefore, miRNAs derived from CSF might serve as more valid biomarkers for brain pathologies than those of other body fluids.

To date only a few studies of miRNA expression in CSF of AD patients have been published. In most of them AD patients were compared to healthy controls, or comparisons of AD patients categorized by different Braak stages were done [18, 20–25]. Even less is known about the differences in CSF miRNA levels between AD and other types of dementia.

Bekris et al. [13] investigated miRNA expression by qPCR in patients with AD compared to both healthy controls and patients with other neurodegenerative diseases. They found miR-15a elevated in the hippocampus and plasma of AD patients where the level was positively correlated with plaque scores. In CSF no expression differences were identified. Burgos et al. [26] profiled miRNAs in CSF and serum by Next Generation Sequencing from patients with AD, Parkinson’s disease (PD) and neurologically healthy controls. Numerous deregulated miRNAs between AD and controls in both CSF and serum were found, whereas only a handful of miRNAs was deregulated between patients with AD and PD. In this study, the expression of several miRNAs in CSF was correlated with Braak stages and tangle scores, including miR-9-3p, for which the level decreased with disease progression. Galimberti et al. [27] profiled miRNAs by qPCR in serum and CSF of AD patients compared to inflammatory and non-inflammatory neurological controls as well as patients with frontotemporal dementia (FTD). They reported down-regulation of miR-125b and miR-26b in CSF of AD patients compared to non-inflammatory controls.

The limited number of studies comparing miRNA CSF levels in AD patients versus patients with other types of dementia and their opposing results justifies the need for additional studies to investigate the utility of miRNAs as biomarkers in a clinical relevant setup.

The purpose of this exploratory investigation was to analyze the expression of miRNAs by RT-qPCR in CSF and blood from 10 patients diagnosed with Alzheimer’s disease and 10 patients diagnosed with either vascular dementia, frontotemporal dementia or dementia with Lewy bodies in order to identify potential miRNA biomarker candidates with the ability to separate AD from other types of dementia using a clinically relevant setup.

## Methods

### Patient material and diagnostic evaluation

Patients who went through diagnostic evaluation at the Memory Clinic, Department of Neurology, Nordsjællands Hospital, from May to December 2014, were asked to participate. All patients gave written and oral informed consent in accordance with the project approval from the Danish Research Ethics Committee (project ID: H-2-2013-069). After completed diagnostic evaluation the study population consisted of 20 patients: one group of AD patients (*n* = 10), and one control group of patients with other types of dementia, including vascular dementia (*n* = 4), frontotemporal dementia (*n* = 4), and dementia with Lewy bodies (*n* = 2).

Diagnoses were based on patient interviews with supplemental reports from relatives/caregivers, physical and neurological examination, evaluation of psychiatric symptoms (if relevant), assessment of activities of daily living, and cognitive testing by experienced specialist neuropsychologists, whose test collection included Mini-Mental State Examination (MMSE) and Addenbrooke’s Cognitive Examination (ACE). In addition, all patients went through paraclinical testing with standard blood examinations, lumbar puncture and Computer Tomography (CT) scan of the brain. If necessary, the CT scan was supplemented by Magnetic Resonance Imaging (MRI) of the brain to evaluate vascular abnormalities and/or hippocampal atrophy. In addition, all patients underwent functional imaging performed with Fluorodeoxyglucose Positron Emission Tomography (^18^F-FDG-PET) to measure glucose metabolism in specific brain areas. Patients with symptoms of dementia with Lewy bodies were scanned with a Dopamine Active Transporter (DAT) scan of the brain to verify low levels of dopamine in the basal ganglia.

Lumbar punctures were done by sterile technique with the use of a 0.7 mm spinal needle when the patients attended their second visit at the clinic two weeks after referral. At least 7 ml CSF were collected from each patient. Blood samples were drawn immediately after the lumbar puncture. CSF samples were analyzed for glucose, protein, red and white blood cell count, oligoclonal IgG bands, IgG index, total tau, phospo-tau and Aβ1-42 as well as CSF/serum albumin ratio using the routine tests at the Department of Clinical Biochemistry. At the same time 1 ml CSF and 1 ml blood from each patient were collected in EDTA tubes for miRNA analysis.

Final diagnoses were made jointly by several specialist neurologists based on all clinical evaluations and paraclinical test results. For the clinical diagnosis of AD the NINCDS-ADRDA criteria for research [28] were used. Only patients who met the criteria for probable AD dementia with evidence of the AD pathophysiological process were included. All patients in the AD group were sporadic AD patients with a mean age of 70 years. The diagnosis of vascular dementia was made according to the NINDS-AIREN criteria [29], and the clinical diagnoses of frontotemporal dementia and dementia with Lewy bodies were made according to the McKhann criteria [30] and McKieth criteria [31], respectively. Table 1 shows the demographic data of the 20 included patients.

**Table 1:** Demographic data of the patients included

Variable	Control group (range)	AD group (range)	Mean difference (CI)	*p*
Number	10	10		
Gender (female/male)	4/6	6/4		n.s.
Age (years)	69.4 (54–79)	70.0 (56–82)	0.6 (−8.7;9.8)	n.s.
Duration (years)	2.8 (1.0-5.5)	2.3 (1.5-4.0)	−0.5 (−1.9;0.9)	n.s.
MMSE	24.1	21.4	−2,7 (−8.1;2.7)	n.s.
ACE	75.4	67.3	−8.1 (−18.0;1.7)	n.s.
Aβ1-42 [pg/ml CSF]	561.4 (315–865)	391.6 (242–755)	−169.8 (−330.0;-9.6)	0.04
Total tau [pg/ml CSF]	481.4 (228–1200)	540.6 (371–742)	59.2 (−223.3;341.7)	n.s.
P-tau [pg/ml CSF]	60.1 (31–148)	82.6 (55–110)	22.5 (−3.2;48.2)	n.s.

*Control group* patients diagnosed with vascular dementia (*n* = 4), frontotemporal dementia (*n* = 4), and dementia with Lewy bodies (*n* = 2). *AD group* patients all diagnosed with Alzheimer’s dementia. *MMSE* Mini-Mental State Examination score, *ACE* Addenbrooke’s Cognitive Examination score. *Mean difference [CI]* difference of mean values between groups and 95 % confidence interval of the difference. *p* p-values of difference calculated by two-sided t-tests or Fisher’s exact test (Gender), *n.s*. nonsignificant (*p* > 0.05)

### Samples for miRNA detection

For miRNA detection, CSF and blood from each patient were centrifuged immediately after collection and the cell-free fractions were stored at −80.0 °C. All further sample preparations and experiments were conducted by Exiqon A/S, Denmark. Total RNA was extracted from 200 μl CSF and 200 μl plasma using spin column chromatography (miRCURY™ RNA isolation kit for biofluids).

### miRNA real-time qPCR

Ten microliters of RNA was reverse transcribed in 50-μl reactions using the miRCURY LNA™ Universal RT miRNA PCR, Polyadenylation and cDNA synthesis kit (Exiqon). cDNA was diluted 50 times and assayed in 10-μl PCR reactions. Each miRNA was assayed once by quantitative polymerase chain reactions (qPCR) on the miRNA Ready-to-Use PCR, Human panel I containing 372 specific miRNA primers. Negative controls were performed and profiled like the samples. Both RNA (Sp2, Sp4, Sp5, and Sp6) and DNA (Sp3) spike-in controls were added to the panel. Amplification was performed in a LightCycler® 480 Real-Time PCR System (Roche). Amplification curves were analyzed using the Roche LC software, both for determination of Ct values and for melting curve analysis.

### Data analysis and normalization

The amplification efficiency was calculated by using LinReg software. All assays were inspected for distinct temperature melting curves, which were checked to be within known specifications for the assay. Only miRNAs detected with Ct < 37 and, in addition, 3 Ct values less than the negative control were included in the data analysis. Data that did not pass these criteria were omitted from any further analysis. Thirty-two miRNAs were detected in all CSF samples, and 115 miRNAs were detected in all blood samples. Using NormFinder [32], the best normalizer was found to be the average Ct value of miRNAs detected in all samples of CSF and blood, respectively, and these global means were used for normalization (relative expression level = REL = 2^-∆Ct^) [33]. Both RNA (Sp6) and DNA (Sp3) spike-in controls showed steady levels across all samples indicating accurate RT reaction and qPCR.

### Statistics

The data of REL or log (REL) did not fit the normal distribution. Therefore, a two-sided non-parametric Mann–Whitney test was performed for statistical analyses (SPSS software) to compare medians (missing values were excluded). *P* values less than 0.05 were considered significant.

To analyze the predictive power of each miRNA with respect to categorizing patients as having either AD or another type of dementia, logistic regression analyses with classification accuracies for each miRNA were performed. For this purpose, a leave-one-out (LOO) cross-validation procedure (R Statistics) was applied (missing values were set to zero).

*P* values for categorical variables were calculated by Fisher’s exact test, and *p* values for comparisons of age, cognitive test score, and CSF routine markers were calculated by student’s *t*-test.

Because of the high number of tests performed, all *P* values were finally corrected for multiple testing with the Benjamini-Hochberg procedure.

## Results

### miRNA distribution in body fluids

**Fig. 1:**
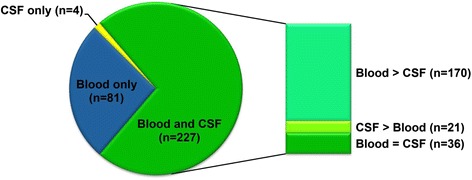
Distribution of the 312 miRNAs detected in CSF and blood of all patients. *Blood > CSF*: miRNAs detected more frequently in blood samples than in CSF samples. *CSF > blood*: miRNAs detected more frequently in CSF samples than in blood samples. *Blood = CSF*: miRNAs detected with equal frequency in blood and CSF samples

Overall, 312 miRNAs were detected in CSF or blood in our study population. Of these, 227 miRNAs occurred in both CSF and blood, 81 miRNAs occurred in blood only, and a minor proportion of 4 miRNAs was found exclusively in the CSF. Among the miRNAs detected in both CSF and blood, some miRNAs were detected predominantly in the CSF and others predominantly in the blood of all patients (Fig. 1)

### miRNAs in CSF

A total of 231 different miRNAs were detected in the CSF. Only 32 different miRNAs were detected in all of the 20 CSF samples. On average, 105 miRNAs were detected in each patient, equally distributed among AD patients and control patients with other types of dementia. We found no significant difference in total amount (Ct) and number of different miRNAs in the two groups.

**Fig. 2:**
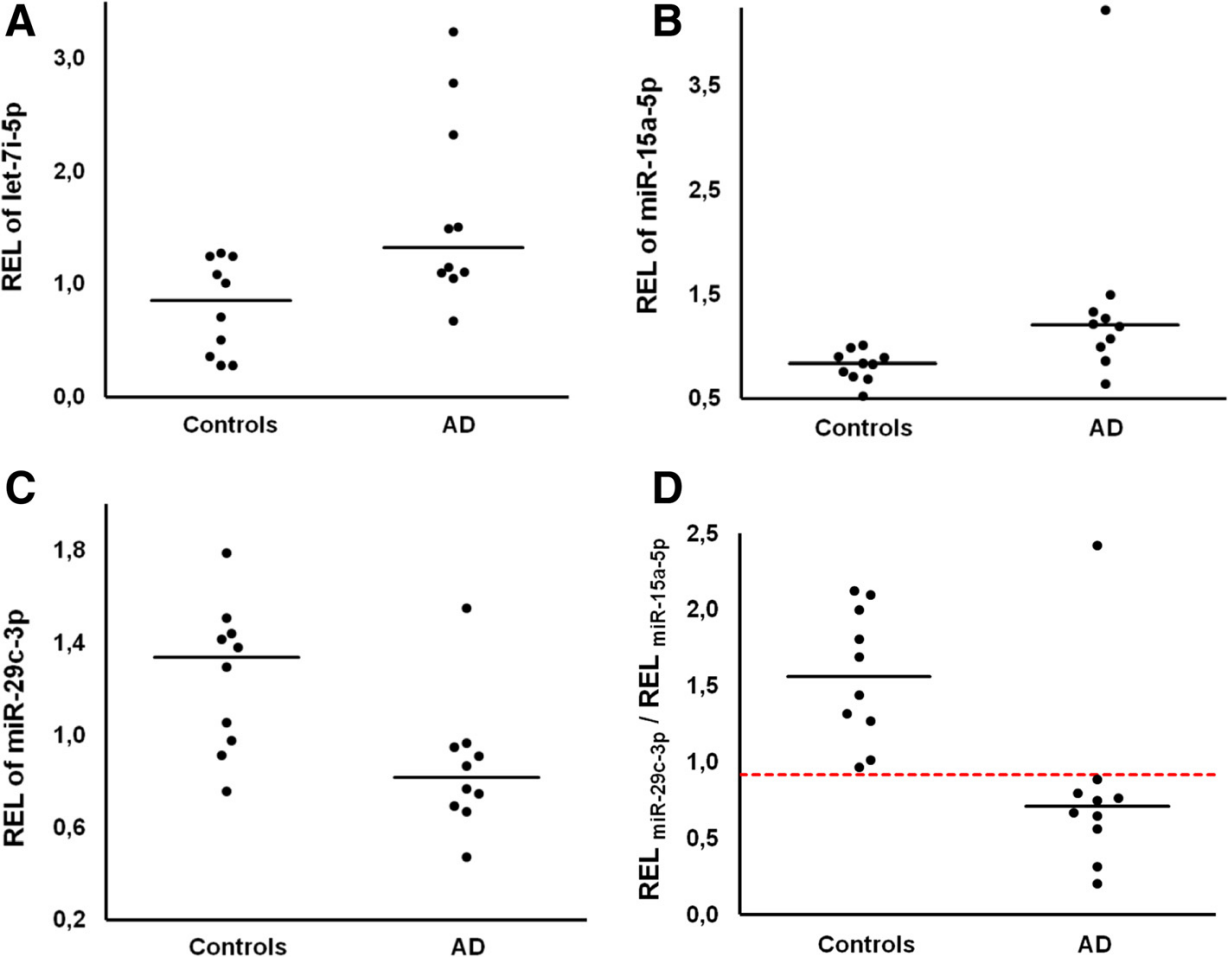
Deregulated miRNAs in CSF of patients with Alzheimer’s disease (*n* = 10) compared to patients with other types of dementia (*n* = 10). Aligned scatter plots show relative expression levels (REL) of let-7i-5p **a**, miR-15a-5p **b**, and miR-29c-3p **c**. Black bars indicate median values. In part **d** the ratio REL_miR-29c-3p_/REL_miR-15a-5p_ is calculated for each patient and plotted in an aligned scatter plot. The red dotted line indicates a cutoff value of 0.92 where AD patients can be separated from control patients with a sensitivity of 90 % and a specificity of 100 %

Fifty-two miRNAs were detected in CSF in at least nine out of ten patients in both groups. When comparing these 52 miRNAs between AD patients and controls, two miRNAs (let-7i-5p and miR-15a-5p) were found significantly up-regulated and one miRNA (miR-29c-3p) was found significantly down-regulated in patients with AD (Table 2, Fig. 2). However, when adjusting all *P* values with the Benjamini-Hochberg procedure for multiple testing, none of them were statistically significant.

**Table 2:** Regulation of the 52 most frequently detected miRNAs in CSF of patients with dementia

miRNA	N_AD_	N_control_	M_AD_ (range)	M_control_ (range)	FC	*p*
miR-15a-5p	10	10	1.20 (0.63–4.22)	0.83 (0.52–1.01)	1.44	0.005
miR-29c-3p	10	10	0.81 (0.47–1.55)	1.33 (0.75–1.78)	0.60	0.009
let-7i-5p	10	10	1.31 (0.67–3.23)	0.85 (0.27–1.27)	1.54	0.019
miR-22-3p	10	9	0.83 (0.20–1.75)	1.35 (0.83–2.01)	0.62	n.s.
miR-24-3p	9	10	0.91 (0.19–1.23)	1.17 (0.81–1.94)	0.78	n.s.
miR-185-5p	10	9	1.35 (0.39–5.13)	0.62 (0.30–1.45)	2.16	n.s.
miR-27b-3p	10	10	0.95 (0.49–1.48)	1.21 (0.63–1.87)	0.78	n.s.
miR-142-3p	9	9	1.19 (0.27–4.31)	0.86 (0.26–1.83)	1.38	n.s.
let-7 g-5p	10	10	1.09 (0.55–2.75)	0.79 (0.39–2.19)	1.37	n.s.
miR-23a-3p	10	10	0.92 (0.53–1.55)	1.11 (0.62–2.04)	0.82	n.s.
miR-23b-3p	10	10	1.00 (0.38–1.24)	1.05 (0.60–2.55)	0.94	n.s.
miR-130a-3p	10	10	0.82 (0.41–1.56)	1.22 (0.42–2.13)	0.67	n.s.
let-7c-5p	10	10	0.88 (0.37–2.21)	1.15 (0.35–3.09)	0.76	n.s.
miR-150-5p	10	9	0.89 (0.23–5.44)	0.82 (0.37–1.68)	1.09	n.s.
miR-361-5p	9	9	0.95 (0.47–1.32)	1.00 (0.70–1.51)	0.94	n.s.
miR-25-3p	10	9	1.00 (0.36–5.49)	0.87 (0.34–1.77)	1.14	n.s.
miR-221-3p	10	10	1.68 (0.15–3.04)	1.22 (0.15–1.70)	1.38	n.s.
miR-29a-3p	10	10	0.96 (0.72–1.30)	1.01 (0.71–1.71)	0.94	n.s.
miR-30a-5p	10	10	1.32 (0.20–2.91)	1.12 (0.37–1.91)	1.17	n.s.
miR-10b-5p	9	10	0.90 (0.26–5.08)	1.04 (0.21–1.70)	0.86	n.s.
let-7a-5p	10	10	0.80 (0.29–2.58)	1.14 (0.65–3.49)	0.70	n.s.
miR-125b-5p	10	10	0.85 (0.62–1.53)	0.98 (0.69–2.77)	0.87	n.s.
miR-16-5p	10	10	0.96 (0.05–9.45)	0.98 (0.56–1.47)	0.98	n.s.
miR-424-5p	10	10	1.19 (0.35–3.38)	0.72 (0.47–2.38)	1.65	n.s.
miR-143-3p	10	10	1.04 (0.45–1.48)	0.92 (0.69–2.32)	1.12	n.s.
miR-19a-3p	9	10	1.14 (0.21–3.68)	1.15 (0.65–1.49)	0.99	n.s.
miR-124-3p	10	9	1.31 (0.26–2.73)	1.21 (0.32–2.08)	1.08	n.s.
let-7b-5p	10	10	1.03 (0.31–3.50)	0.83 (0.45–3.39)	1.23	n.s.
miR-146a-5p	9	10	1.37 (0.23–2.40)	0.91 (0.20–2.69)	1.50	n.s.
let-7d-3p	9	10	1.02 (0.54–1.89)	1.33 (0.62–1.83)	0.76	n.s.
miR-125a-5p	10	9	1.21 (0.32–2.96)	0.71 (0.29–2.68)	1.69	n.s.
miR-27a-3p	9	10	1.14 (0.15–2.81)	1.29 (0.25–3.03)	0.88	n.s.
miR-30d-5p	10	10	1.16 (0.31–2.47)	1.15 (0.34–1.83)	1.01	n.s.
miR-99a-5p	10	10	0.98 (0.57–1.36)	1.07 (0.62–1.93)	0.91	n.s.
miR-145-5p	10	10	1.16 (0.30–1.73)	1.00 (0.47–2.05)	1.15	n.s.
miR-100-5p	10	10	0.92 (0.54–1.88)	1.07 (0.63–1.86)	0.85	n.s.
miR-223-3p	9	10	1.14 (0.86–2.74)	0.78 (0.22–4.81)	1.45	n.s.
miR-320a	10	10	0.94 (0.54–1.62)	1.05 (0.64–1.68)	0.90	n.s.
miR-497-5p	10	10	1.07 (0.51–1.72)	1.08 (0.64–1.81)	0.98	n.s.
miR-373-5p	9	10	1.10 (0.26–2.66)	1.11 (0.30–3.44)	0.98	n.s.
miR-204-5p	10	10	1.05 (0.07–2.30)	0.99 (0.47–2.28)	1.06	n.s.
miR-92a-3p	10	10	0.94 (0.70–3.69)	0.84 (0.47–2.17)	1.12	n.s.
miR-423-5p	10	10	1.11 (0.65–2.27)	0.78 (0.44–2.22)	1.42	n.s.
miR-19b-3p	10	10	1.08 (0.18–2.25)	1.20 (0.21–2.20)	0.90	n.s.
miR-9-5p	9	10	1.25 (0.50–1.97)	1.24 (0.24–3.11)	1.00	n.s.
miR-30c-5p	10	10	1.15 (0.20–2.57)	0.99 (0.37–2.38)	1.15	n.s.
miR-21-5p	10	10	0.89 (0.66–2.29)	1.06 (0.27–1.86)	0.83	n.s.
miR-34a-5p	10	9	0.99 (0.26–2.39)	1.06 (0.46–2.07)	0.94	n.s.
miR-101-3p	10	9	1.11 (0.37–1.48)	0.93 (0.56–2.64)	1.19	n.s.
miR-29b-3p	9	9	1.12 (0.31–1.76)	0.92 (0.49–2.02)	1.22	n.s.
miR-199a-3p	10	10	0.92 (0.58–3.40)	1.16 (0.39–1.67)	0.79	n.s.
miR-9-3p	10	10	1.13 (0.23–2.35)	1.02 (0.28–2.86)	1.09	n.s.

*N*_*AD*_ number of Alzheimer patients in which the miRNA was detected, *N*_*control*_ number of control patients in which the miRNA was detected, *M*_*AD*_ the median of relative expression levels among Alzheimer patients, *M*_*control*_ median of relative expression levels among control patients, *FC* fold change of median values, *p* p-values of difference calculated by two-sided nonparametric Mann–Whitney test (not corrected for multiple testing), *n.s*. nonsignificant (*p* > 0.05)

**Fig. 3:**
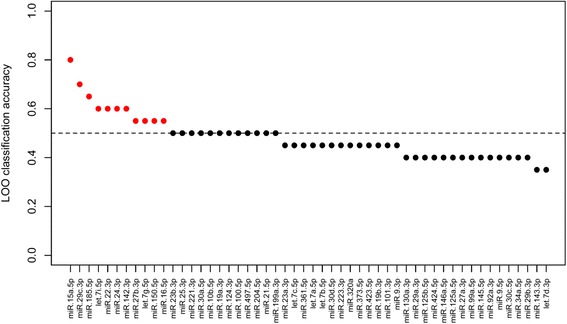
Logistic regression cross-validated classification accuracies based on a leave-one-out procedure (LOO). The 52 most frequently detected miRNAs in CSF are ordered by decreasing magnitude of predictive power. Red color indicates predictors with classification accuracy above random guessing

By combining two of the differentially expressed miRNAs (miR-29c-3p and miR-15a-5p) in a simple ratio model (REL_miR-29c-3p_/REL_miR-15a-5p_) AD patients could be separated from patients with other types of dementia (cutoff value 0.92) with a sensitivity of 90 % and a specificity of 100 % (Fig. 2 part D). To analyze the predictive power of each miRNA in order to categorize patients as having either AD or another type of dementia logistic regression analysis were done for the 52 frequently detected miRNAs in CSF (Fig. 3). The best predictors identified by this procedure were again miR-15a-5p and miR-29c-3p with classification accuracies of 0.8 and 0.7, respectively.

When considering the detected miRNAs in CSF as categorical variables, three miRNAs were found to be expressed more often among AD patients compared to controls (Table 3). This difference was most significant for two miRNA in CSF (miR-199b-5p and miR-22-5p). Both miRNAs were detected in the CSF of 50 % of AD patients and were not detected in the CSF of controls.

**Table 3:** miRNAs detected more often in CSF samples of AD patients compared to patients with other types of dementia

miRNA	N_AD_	N_control_	OR	Sensitivity	Specificity	*p*
miR-199b-5p	5/10	0/10	21	50	100	0.03*
miR-22-5p	5/10	0/10	21	50	100	0.03*
miR-206	6/10	1/10	13.5	60	90	0.06

*N*_*AD*_ number of patients with Alzheimer’s disease in which the miRNA was detected, *N*_*control*_ number of patients with other types of dementia in which the miRNA was detected, *OR* odds ratio, *p* p-value (Fischer’s exact test). Star indicates statistically significance (*p* < 0.05)

### miRNAs in blood

A total of 308 different miRNAs were detected in the blood. Of these, 115 miRNAs were detected in all of the 20 blood samples. On average, 227 miRNAs were detected in each patient. One hundred and sixty-eight miRNAs were detected in at least nine out of 10 patients in each group (Additional file 1). Among these, miR-590-5p (fold change (FC) = 1.35, *p* = 0.002) and miR-142-5p (FC = 1.22, *p* = 0.043) were significantly up-regulated and miR-194-5p (FC = 0.54, *p* = 0.028) was significantly down-regulated in AD patients compared to controls. Again, these *P* values would not pass a Benjamini-Hochberg correction for multiple testing.

When applying logistic regression analyses to blood data we identified 30 miRNAs with classification accuracies above 0.50 (i.e. random guessing), of which miR-590-5p and miR-194-5p had the highest accuracy values (Additional file 2).

No candidates for categorical variables were identified in the blood.

### Blood–brain Barrier Function

As a measure of BBB dysfunction, the albumin CSF/serum concentration quotient (Q_alb_) is a widely accepted indicator [34]. Albumin is a serum protein that is normally prevented from passing into the CSF. However, under certain conditions such as neuroinflammation the BBB may become leaky allowing albumin molecules to pass from the blood. In this study, four out of ten AD patients had elevated Q_alb_ values. Three patients in the control group, one with vascular dementia and two with frontotemporal dementia, also had elevated Q_alb_ as sign of a leaky BBB (Additional file 3).

## Discussion

Cognitive impairment may occur in a wide range of neurological, psychiatric and medical conditions, and the differential diagnosis of AD may be difficult, particularly against patients with other neurodegenerative or vascular brain diseases. Therefore we intended to set up a clinical relevant study design by choosing a non-healthy control group of patients with other types of dementia. Thus, with this exploratory investigation we aimed to identify expression differences of miRNAs in patients diagnosed with Alzheimer’s disease and patients diagnosed with either vascular dementia, frontotemporal dementia or dementia with Lewy bodies in order to identify potential miRNA biomarker candidates with the ability to separate AD from other types of dementia. By carrying out a screening for 372 known miRNA sequences with RT-qPCR we identified a differential expression pattern for let-7i-5p, miR-15a-5p, and miR-29c-3p in the CSF of AD patients and for miR-590-5p, miR-142-5p, and miR-194-5p in the blood of AD patients as compared to patients with other types of dementia.

### Overall miRNA detection

On average, 105 different miRNAs were detected in the CSF of both cases and controls. These findings are consistent with results from other studies, including our own previous study of miRNA detection in CSF of stroke patients [35], in which a slightly smaller number of miRNAs were identified (*n* = 73) in each patient. The average number of detected miRNAs in blood was 227 as compared to 204 in our previous study, and the distribution patterns of miRNAs in blood and CSF in our studies have been very similar (Fig. 1) suggesting a reliable miRNA analysis. Data have been thoroughly checked using negative controls in the RT step and melting curve analysis. Furthermore, the steady levels of DNA and RNA spike-ins indicate good technical performance of our profiling experiment.

### Findings in CSF

In the CSF we identified a differential expression pattern for miR-15a-5p, let-7i-5p, and miR-29c-3p. In our study, miR-15a-5p was up-regulated with a fold change of 1.44 (*p* = 0.005) in the CSF of AD patients. Bekris et al. [13] have previously found elevated levels of miR-15a in plasma and hippocampal tissue of AD patients with a positive correlation to plaque scores. By contrast, Hebert et al. [15] have found miR-15a down-regulated in cerebral cortex of AD patients compared to healthy controls. Interestingly, miR-15a’s regulation of both APP and BACE1 has been validated in various ways, including reporter assay analysis [15, 36]. Since the miRNA-mRNA binding usually results in gene silencing via translational repression, one could argue that the level of miR-15a-5p was expected to be lower in AD patients compared to other types of dementia. However, as the complex pathogenesis of late-onset AD is not limited to an increased Aβ production but also involves impaired Aβ clearance [37], the possible ways for interaction of miR-15a-5p in AD biology are many. Among the list of predicted targets for miR-15a obtained from the miRWalk database [38] we discovered other genes relevant to late-onset AD [39] including CD2-associated protein (CD2AP) which recently has been suggested to mediate the integrity of the BBB [40].

In the CSF of AD patients we also found an up-regulation of let-7i-5p (FC = 1.54, *p* = 0.019) compared to other types of dementia. Burgos et al. [26] reported a positive correlation between the level of let-7i-3p in serum of AD patients and their Braak stages. Even more interesting, Lehmann et al. [20] found let-7b, another member of the let-7 family of miRNAs, elevated in the CSF of AD patients. Through reporter assay experiments and by intrathecal injection of let-7b into the CSF of wild-type mice Lehmann et al. showed that extracellular let-7b activated the RNA-sensning Toll-like receptor 7 (TLR7) in both immune cells and neurons, and that this activation resulted in neuronal cell death. From these experiments the authors hypothesized that extracellular miRNAs like let-7 can contribute to spread neuronal damage from one brain region through activation of extracellular TLR4. Also Let-7i-5p, which was elevated in the CSF of AD patients in our study, has specifically been validated to target TLR7 [41].

We found miR-29c-3p down-regulated in the CSF of AD patients with a FC of 0.60 (*p* = 0.009). This finding is in accordance with the results from Hebert et al. [15] as previously mentioned, who found a significant down-regulation of miR-29a and miR-29b (other members of the miR-29 family) in temporal and cerebellar cortical tissue from sporadic AD patients compared to healthy controls. BACE1 was validated as a target of miR-29a/b by reporter assays, and furthermore the inhibiting effect of miR-29a/b on BACE1 activity was corroborated by gain- and loss of function experiments using HEK293 cells. In mice brain tissue and cell cultures, the suppressing effect of miR-29c-3p (down-regulated in our study) on BACE1 has also been validated by Zong et al. [42] by various methods. Most recently, Lei et al. [11] found down-regulation of miR-29c in brain tissues from sporadic AD patients in whom the miR-29c level was negatively correlated with BACE1 mRNA level. In contrast to these experiments and to our results (Table 2), Kiko et al. [25] have found significant up-regulation of miR-29a and miR-29b in the CSF of AD patients compared to healthy control subjects. In this context, it has, however, not been clarified whether each member of the miR-29 family of miRNAs is expected to be regulated in the same direction.

When considering the detected miRNAs in CSF as categorical variables, three miRNAs were found to be expressed more often in the CSF of AD patients compared to controls (Table 3), namely miR-199b-5p, miR-22-5p, and miR-206. Taking into account the small study population and the large number of observations in our qPCR experiment, however we cannot exclude the possibility of randomness in this observation. The expression of miR-206 has been linked to the regulation of brain-derived neurotrophic factor (BDNF), a regulator of synaptic plasticity and memory, which is known to be deficient in AD brains. Lee et al. [43] found up-regulation of miR-206 in human temporal cortex of AD patients and in transgenic AD mice, and validated its repression of BDNF by northern blotting and qPCR. Similarly, Tian et al. [44] reported an up-regulation of miR-206 in hippocampal tissue, cerebrospinal fluid, and plasma of embryonic APP/PS1 transgenic mice, and found that miR-206 depressed the expression of BDNF.

To our knowledge, the possible functions miR-199b-5p and miR-22-5p in relation to AD and other dementias are unknown.

### Findings in blood

Despite the large number of frequently detected miRNAs in the blood (*n* = 168), only three miRNAs (miR-590-5p, miR-142-5p, and miR-194-5p) were identified as being differential expressed between AD patients and controls.

The expression of miR-590-5p, which was up-regulated in the blood of AD patients in our study, has previously been reported up-regulated in plasma of patients with vulnerable coronary artery disease [45]. The antisense string, miR-590-3p, has been investigated in an extensive study involving 274 patients with frontotemporal dementia, 287 patients with AD, and 344 non-demented age-matched controls [46]. In this study the expression of miR-590-3p was decreased in mononuclear cells in peripheral blood of AD patients compared to non-demented control subjects, and its target hnRNPA1, a protein involved in the maturation of APP, was up-regulated. The study is not directly comparable to our experiment mainly because of the different cells and body fluids examined, and therefore the meaning of the oppositely directed regulation of miR-590 found in these two studies remains unclear.

With regard to miR-142-5p, which was up-regulated in the blood of AD patients in our study, the only validated target is nuclear factor, erythroid 2-like 2 (NRF2). This transcription factor regulates several genes that encode proteins involved in responses to injury and inflammation which includes the production of free radicals [47]. Results from other clinical studies are in some ways different from ours. Cogswell et al. [18] found miR-142-5p down-regulated in the CSF of AD patients compared to controls, and Kumar et al. [48] found the antisense string, miR-142-3p, down-regulated in plasma of AD patients compared to both MCI patients and healthy controls.

In our study, miR-194-5p was down-regulated in the blood of AD patients. We have no knowledge from previous studies or from literature searches about the role and function of this miRNA in relation to AD or other types of dementia.

Another miRNA, miR-107, which we found non-significantly down-regulated in the blood of AD patients, but which had a relatively high classification accuracy (Additional file 2), has interestingly been reported down-regulated in the blood of AD patients in two previous studies [49, 50]. This miRNA is known to target BACE1, and the level of miR-107 was previously found down-regulated in AD brain tissue, suggesting a role for miR-107 in the pathogenesis of AD [10].

In summary, most of the deregulated miRNAs that we have identified in both CSF and blood can be linked to relevant target molecules, although the overall picture of our findings is still unclear and warrants further elucidation. Existing studies differ in design and extraction methods making it difficult to compare results and to gain a complete overview of miRNAs affected by AD.

### Best biomarker candidates

A biomarker measured in the blood is preferable to more invasive alternatives. Blood samples from patients are obtained easily at a low cost and at a low risk of adverse effects. However, blood is a systemic fluid which composition may reflect processes in other tissues or organs besides the brain. CSF, on the other hand, which is protected by the blood–brain barrier, ought to be a reliable biomarker source for neurodegenerative diseases in the central nervous system. In our study, the results from miRNA expression analysis in the CSF seem more interesting compared to blood data in various ways. Firstly, an equal number of miRNAs were differentially expressed in blood and CSF despite the four times larger number of frequently detected miRNAs in the blood. Secondly, we find it interesting that the three differential expressed miRNAs in CSF (miR-29c-3p, miR15a-5p, and let-7i-5p) could all be related to AD relevant targets like APP and BACE1.

miR-29c-3p and miR-15a-5p had the highest classification accuracies separately, and by combing them in a simple ratio model (REL_miR-29c-3p_/REL_miR-15a-5p_) AD patients could be separated from patients with other types of dementia (cutoff value 0.92) with a sensitivity of 90 % and a specificity of 100 %. This ratio model is of course associated with statistical uncertainty due to our small population size, and should therefore only be seen as a proposal for the use of these markers in future studies, which are highly needed. Logistic regression models that combines more than one miRNA is another approach, but again in this small sized pilot study it is impossible to select a single best model according to the low stability proportion of each predictor and none of our *P* values were strong enough to be considered significant after correction for multiple testing.

### The impact of the BBB

Although the CSF is isolated from the rest of the circulation by the blood–brain barrier, under certain conditions, such as inflammation, the barrier can become leaky allowing molecules to pass from the blood. To measure the BBB function we calculated CSF/serum albumin ratios (Q_alb_) in the 20 patients included. In this study, four out of ten AD patients had elevated Q_alb_ values. Three patients in the control group also had elevated Q_alb_ values indicating a leaky BBB. Thus, in those patients the presence of miRNAs in the CSF could simply be a result of contamination from the systemic circulation. When examining the expression of miR-22-5p, miR-199b-5p and miR-206 (detected in the CSF with a frequency of 50 % in the AD group and 0 % in the control group) we found no association between the expression of these miRNAs in the blood and CSF of patients with an elevated Q_alb_, suggesting that their presence in CSF does not seem to be explained by a leak in BBB. Also, we did not find an obvious association between the expression of other miRNAs in blood and CSF, although a direct correlation analysis between the levels in blood and CSF was prevented due to the small size of our study population. Lastly we noticed that some miRNAs which have been characterized as brain specific, such as miR-124 and miR-9, were detected predominantly in the CSF, further indicating that the expression of miRNAs in the two body fluids is individually regulated and probably unaffected by small disturbances in the BBB function. Findings by Bekris et al. [13] and Burgos et al. [26] support this notion since they also did not find any correlation between the expression of individual miRNAs in CSF and blood in their studies. Neither of these two studies included other specific measures to evaluate the integrity of the BBB whereas we calculated the Q_alb_ as a measure of BBB dysfunction [34]. The findings taken together should, however, be interpreted with caution and further studies are warranted to clarify the origin of miRNAs in the CSF and their transport across the BBB.

## Conclusions

Detection of miRNA expression profiles in blood and in particular CSF of patients diagnosed with different types of dementia is feasible and it seems that several expressional differences between AD and other dementia types do exist in a clinically relevant setup like the present. In this explorative pilot study, the deregulated miRNAs in CSF of AD patients compared to a relevant non-healthy control group may be associated with target genes related to AD pathology, including APP and BACE1, which suggests that miRNAs are interesting candidates for AD biomarkers in the future.

Clinical validation of our findings in a larger scale is obviously needed. We are currently working on a validation study with larger study groups that will enable subgroup analyses between the different types of dementia and also allow us to make stronger model assumptions based on combinations of several miRNAs as diagnostic biomarkers of the different types of dementia.

### Ethics approval and consent to participate

A statement on ethics approval and consent to participate is included in the methods section.

### Consent for publication

All included patients gave written and oral informed consent for publication.

### Availability of data and materials

The datasets supporting the conclusions of this article are included within the article and its additional files.

## Additional files

Additional file 1: Regulation of the 168 most frequently detected miRNAs in blood of patients with dementia. *N*_*AD*_ number of Alzheimer patients in which the miRNA was detected, *N*_*control*_ number of control patients in which the miRNA was detected, *M*_*AD*_ the median of relative expression levels among Alzheimer patients, *M*_*control*_ median of relative expression levels among control patients, *FC* fold change of median values, *p* p-values of difference calculated by two-sided nonparametric Mann–Whitney test (not corrected for multiple testing), *n.s*. nonsignificant (*p* > 0.05). (PDF 412 kb) Additional file 2: Logistic regression cross-validated classification accuracies based on a leave-one-out procedure (LOO). The 168 most frequently detected miRNAs in blood are ordered by decreasing magnitude of predictive power. Red color indicates predictors with classification accuracy above random guessing. (PDF 146 kb) Additional file 3: Estimation of the blood brain barrier dysfunction in patients with dementia based on albumin CSF/serum concentration quotients. *AD* Alzheimer’s disease, *DLB* dementia with Lewy bodies, *VAD* vascular dementia, *FTD* frontotemporal dementia. Q_alb_ albumin CSF/serum concentration quotient. Q_alb limit_ age dependent upper limit of albumin CSF/serum concentration quotient calculated by the formula ${Q}_{alb}=\left(4+\frac{age}{15}\right)$. *BBB* blood brain barrier. (PDF 447 kb)

## Abbreviations

miRNAs: MicroRNAs
CSF: cerebrospinal fluid
AD: Alzheimer’s disease
Aβ1-42: amyloid beta
mRNA: messenger RNA
BACE1: amyloid precursor protein cleaving enzyme 1
BBB: blood–brain barrier
PD: Parkinson’s disease
FTD: frontotemporal dementia
DLB: dementia with Lewy bodies
VAD: vascular dementia
MMSE: Mini-Mental State Examination
ACE: Addenbrooke’s Cognitive Examination
CT: Computer Tomography
MRI: Magnetic Resonance Imaging
F-FDG-PET: Fluorodeoxyglucose Positron Emission Tomography
DAT: Dopamine Active Transporter
qPCR: quantitative polymerase chain reactions
REL: relative expression level
LOO: leave-one-out cross-validation procedure
FC: fold change
Q_alb_: albumin CSF/serum concentration quotient
