# Supplementary material for: miRNA expression profiles in cerebrospinal fluid and blood of patients with Alzheimer’s disease and other types of dementia – an exploratory study
Source: Transl Neurodegener. 2016 Mar 15;5:6. doi: 10.1186/s40035-016-0053-5 (PMC4791887; doi:10.1186/s40035-016-0053-5)
Supplement: Additional file 1: — Regulation of the 168 most frequently detected miRNAs in blood of patients with dementia. N AD number of Alzheimer patients in which the miRNA was detected, N control number of control patients in which the miRNA was detected, M AD the median of relative expression levels among Alzheimer patients, M control median of relative expression levels among control patients, FC fold change of median values, p p-values of difference calculated by two-sided nonparametric Mann–Whitney test (not corrected for multiple testing), n.s. nonsignificant (p > 0.05). (PDF 412 kb) [file 40035_2016_53_MOESM1_ESM.pdf]

Additional file 1. Regulation of the 168 most frequently detected miRNAs in blood of patients with dementia

miRNA	N_{AD}	$N_{control}$	M_{AD} (range)	$M_{control}$ (range)	FC	p
miR-590-5p	10	10	1.19 (0.83-1.88)	0.88 (0.52-1.03)	1.35	0.002
miR-194-5p	9	10	0.74 (0.49-1.32)	1.36 (0.48-2.62)	0.54	0.028
miR-142-5p	10	10	1.13 (0.37-2.08)	0.92 (0.43-1.66)	1.22	0.043
miR-30e-5p	10	10	0.91 (0.82-1.19)	1.06 (0.85-1.28)	0.85	n.s.
miR-425-3p	10	9	0.85 (0.35-1.62)	1.20 (0.51-1.79)	0.70	n.s.
miR-338-3p	10	10	1.21 (0.51-2.30)	0.71 (0.45-1.63)	1.71	n.s.
let-7f-5p	10	10	1.07 (0.28-1.51)	1.27 (0.72-2.11)	0.83	n.s.
miR-18b-5p	10	10	1.10 (0.85-1.74)	0.95 (0.62-1.20)	1.14	n.s.
miR-186-5p	10	10	0.84 (0.32-1.53)	1.19 (0.57-2.36)	0.71	n.s.
miR-107	10	10	0.95 (0.47-1.21)	1.09 (0.88-1.42)	0.87	n.s.
let-7c-5p	10	10	0.97 (0.28-1.40)	1.15 (0.72-1.94)	0.84	n.s.
miR-27a-3p	10	10	1.09 (0.55-1.77)	0.88 (0.63-1.30)	1.24	n.s.
miR-377-3p	9	10	1.18 (0.29-3.77)	0.72 (0.16-1.88)	1.63	n.s.
miR-139-5p	10	10	1.27 (0.28-2.42)	0.90 (0.33-1.58)	1.40	n.s.
miR-628-3p	9	9	0.97 (0.30-2.89)	0.51 (0.34-1.28)	1.89	n.s.
miR-181a-5p	10	10	0.81 (0.61-1.26)	1.03 (0.66-2.30)	0.78	n.s.
miR-486-5p	10	10	1.00 (0.37-1.37)	1.40 (0.48-2.18)	0.71	n.s.
miR-505-3p	9	9	0.70 (0.37-1.02)	0.84 (0.58-1.49)	0.82	n.s.
miR-215-5p	9	9	0.61 (0.39-1.16)	0.99 (0.37-2.07)	0.61	n.s.
miR-766-3p	9	10	0.94 (0.64-1.30)	0.87 (0.48-1.03)	1.07	n.s.
miR-345-5p	10	10	1.01 (0.63-2.64)	0.88 (0.54-1.45)	1.15	n.s.
miR-28-5p	9	10	0.99 (0.59-1.37)	0.78 (0.44-1.51)	1.27	n.s.
miR-545-3p	9	10	1.02 (0.55-4.93)	0.58 (0.31-2.49)	1.77	n.s.
miR-155-5p	9	10	0.71 (0.37-1.50)	0.88 (0.44-2.16)	0.80	n.s.
miR-15b-5p	10	10	0.90 (0.25-1.93)	1.15 (0.42-2.01)	0.77	n.s.
miR-660-5p	10	10	1.12 (0.53-2.02)	1.02 (0.52-1.29)	1.09	n.s.
miR-629-5p	9	10	0.74 (0.32-1.72)	0.80 (0.54-2.94)	0.92	n.s.
let-7a-5p	10	10	0.98 (0.52-1.37)	0.97 (0.78-2.03)	1.01	n.s.
miR-98-5p	10	10	0.89 (0.26-1.48)	1.06 (0.73-2.42)	0.83	n.s.
miR-183-5p	10	10	1.44 (0.31-4.81)	0.95 (0.31-2.53)	1.50	n.s.
miR-151a-3p	10	10	0.98 (0.49-1.29)	1.14 (0.74-1.47)	0.85	n.s.
let-7b-5p	10	10	0.80 (0.49-2.02)	1.23 (0.59-2.44)	0.65	n.s.
miR-885-5p	10	9	0.85 (0.28-2.05)	1.06 (0.06-5.20)	0.79	n.s.
let-7i-5p	10	10	0.83 (0.56-1.88)	1.13 (0.60-1.97)	0.73	n.s.
miR-199a-5p	10	10	1.44 (0.35-2.06)	0.93 (0.46-1.61)	1.54	n.s.

miR-130b-3p	10	10	1.05 (0.72-1.52)	0.89 (0.63-1.43)	1.16	n.s.
miR-148a-3p	10	10	1.03 (0.50-1.18)	0.99 (0.64-1.78)	1.04	n.s.
miR-15a-5p	10	10	0.74 (0.36-1.98)	1.15 (0.62-2.18)	0.64	n.s.
miR-26b-5p	10	10	1.05 (0.29-1.74)	1.12 (0.73-1.71)	0.93	n.s.
miR-197-3p	9	10	0.96 (0.62-1.07)	0.84 (0.54-1.14)	1.13	n.s.
miR-122-5p	10	10	1.15 (0.24-2.72)	1.15 (0.09-6.21)	0.99	n.s.
miR-21-5p	10	10	1.02 (0.68-1.28)	1.01 (0.74-1.54)	1.00	n.s.
let-7d-5p	10	10	0.99 (0.51-1.41)	1.02 (0.73-1.75)	0.96	n.s.
miR-152-3p	9	10	0.86 (0.48-1.14)	0.91 (0.51-1.79)	0.94	n.s.
miR-337-5p	9	9	0.83 (0.34-2.77)	0.81 (0.20-2.21)	1.02	n.s.
miR-18a-5p	10	10	1.01 (0.67-1.97)	0.96 (0.68-1.21)	1.06	n.s.
miR-424-5p	10	10	1.04 (0.23-2.90)	1.02 (0.23-1.74)	1.01	n.s.
miR-375	9	9	0.77 (0.38-1.66)	0.68 (0.33-3.29)	1.13	n.s.
miR-497-5p	10	10	1.03 (0.64-2.38)	0.97 (0.30-1.95)	1.05	n.s.
miR-223-3p	10	10	1.05 (0.69-1.62)	1.01 (0.59-1.31)	1.03	n.s.
miR-106a-5p	10	10	1.04 (0.74-1.63)	0.90 (0.66-1.42)	1.16	n.s.
miR-210-3p	10	10	1.05 (0.62-1.74)	0.96 (0.58-1.54)	1.08	n.s.
miR-382-5p	10	10	1.08 (0.57-5.44)	0.76 (0.22-2.50)	1.42	n.s.
miR-92a-3p	10	10	0.99 (0.45-1.79)	1.17 (0.61-1.87)	0.84	n.s.
miR-27b-3p	10	10	0.99 (0.79-1.96)	0.88 (0.47-1.45)	1.11	n.s.
miR-192-5p	10	10	0.88 (0.57-2.22)	1.27 (0.32-2.56)	0.69	n.s.
miR-146b-5p	9	10	0.80 (0.28-1.56)	1.03 (0.31-1.37)	0.77	n.s.
miR-19b-3p	10	10	1.03 (0.82-1.51)	0.95 (0.65-1.34)	1.08	n.s.
miR-30d-5p	10	10	1.03 (0.87-1.40)	0.93 (0.82-1.18)	1.10	n.s.
miR-423-5p	10	10	0.94 (0.43-1.75)	1.06 (0.60-2.20)	0.88	n.s.
miR-1	9	9	0.99 (0.24-2.70)	0.93 (0.19-5.69)	1.06	n.s.
miR-17-5p	10	10	0.99 (0.50-1.42)	1.20 (0.40-1.66)	0.82	n.s.
miR-323a-3p	9	10	0.89 (0.20-4.43)	0.87 (0.19-3.27)	1.02	n.s.
miR-222-3p	10	10	0.97 (0.67-1.24)	1.07 (0.74-1.41)	0.90	n.s.
miR-19a-3p	10	10	1.04 (0.72-1.43)	1.01 (0.55-1.33)	1.02	n.s.
miR-101-3p	10	10	1.06 (0.69-1.65)	0.88 (0.62-1.52)	1.19	n.s.
miR-130a-3p	10	10	1.07 (0.69-1.39)	0.88 (0.61-1.65)	1.20	n.s.
miR-409-3p	10	10	1.18 (0.38-1.48)	0.93 (0.16-3.87)	1.26	n.s.
miR-598-3p	9	10	0.86 (0.53-3.21)	0.82 (0.49-1.97)	1.04	n.s.
miR-199a-3p	10	10	0.98 (0.80-1.19)	1.03 (0.68-1.57)	0.94	n.s.
miR-96-5p	9	10	0.77 (0.10-4.59)	1.56 (0.18-3.07)	0.49	n.s.
miR-25-3p	10	10	1.01 (0.38-1.54)	1.14 (0.52-1.88)	0.88	n.s.
miR-484	10	10	1.00 (0.86-1.27)	0.94 (0.71-1.51)	1.06	n.s.
miR-20b-5p	9	10	0.55 (0.36-2.61)	1.13 (0.32-2.22)	0.49	n.s.

miR-376c-3p	9	10	1.18 (0.34-2.23)	0.87 (0.23-3.01)	1.35	n.s.
miR-335-5p	10	10	0.90 (0.50-2.25)	1.04 (0.34-1.69)	0.86	n.s.
miR-495-3p	10	10	1.11 (0.26-1.54)	1.02 (0.37-2.62)	1.08	n.s.
miR-195-5p	9	10	1.02 (0.56-1.54)	0.89 (0.29-1.48)	1.14	n.s.
miR-326	9	10	0.88 (0.34-1.61)	0.89 (0.32-1.55)	0.98	n.s.
miR-146a-5p	10	10	1.04 (0.61-1.36)	0.94 (0.63-1.48)	1.10	n.s.
miR-143-3p	10	10	1.42 (0.38-1.77)	1.07 (0.40-1.61)	1.33	n.s.
miR-193b-3p	9	10	0.83 (0.14-3.30)	0.70 (0.16-8.74)	1.17	n.s.
miR-133b	9	10	1.09 (0.32-4.01)	0.86 (0.07-3.15)	1.25	n.s.
miR-331-3p	10	10	1.23 (0.33-1.74)	0.98 (0.58-1.90)	1.24	n.s.
miR-106b-5p	10	10	1.08 (0.86-1.54)	0.92 (0.45-2.08)	1.17	n.s.
miR-99b-5p	10	10	1.03 (0.60-1.57)	1.03 (0.36-2.22)	0.99	n.s.
miR-30c-5p	10	10	1.04 (0.42-1.64)	0.96 (0.83-1.97)	1.07	n.s.
miR-625-3p	9	10	0.74 (0.27-1.98)	0.89 (0.45-2.46)	0.83	n.s.
miR-100-5p	9	10	0.83 (0.27-1.55)	0.74 (0.56-2.15)	1.11	n.s.
miR-10a-5p	9	9	0.94 (0.38-1.85)	0.84 (0.37-1.23)	1.12	n.s.
miR-30a-5p	9	10	1.04 (0.46-1.22)	0.87 (0.34-1.95)	1.19	n.s.
miR-425-5p	10	10	1.00 (0.46-1.34)	1.02 (0.79-1.41)	0.98	n.s.
miR-454-3p	9	10	0.85 (0.41-1.37)	0.91 (0.35-1.97)	0.92	n.s.
miR-539-5p	9	9	0.57 (0.28-4.13)	1.01 (0.16-4.58)	0.56	n.s.
miR-33a-5p	10	10	1.15 (0.60-1.69)	0.99 (0.23-2.31)	1.16	n.s.
miR-132-3p	10	10	1.13 (0.57-1.64)	0.94 (0.58-1.95)	1.19	n.s.
miR-324-3p	10	10	1.12 (0.67-1.31)	0.97 (0.76-1.40)	1.15	n.s.
miR-200a-3p	9	9	0.60 (0.38-5.46)	1.22 (0.20-3.45)	0.49	n.s.
miR-99a-5p	10	10	0.86 (0.54-1.99)	1.08 (0.46-2.50)	0.79	n.s.
miR-125b-5p	10	10	0.92 (0.51-2.77)	1.02 (0.16-2.02)	0.91	n.s.
let-7g-5p	10	10	0.94 (0.63-1.65)	1.03 (0.60-1.54)	0.91	n.s.
miR-29a-3p	10	10	0.99 (0.60-1.65)	0.87 (0.65-1.77)	1.13	n.s.
miR-376a-3p	9	10	0.99 (0.30-3.26)	1.18 (0.33-3.43)	0.83	n.s.
miR-22-5p	10	10	0.83 (0.67-1.84)	1.12 (0.65-1.36)	0.74	n.s.
miR-191-5p	10	10	1.04 (0.49-1.54)	1.04 (0.62-1.41)	0.99	n.s.
miR-141-3p	9	9	0.97 (0.53-2.02)	0.53 (0.23-2.83)	1.82	n.s.
miR-376b-3p	9	10	1.01 (0.36-2.18)	0.91 (0.20-2.45)	1.11	n.s.
miR-196b-5p	9	9	0.82 (0.16-1.48)	0.84 (0.30-1.68)	0.97	n.s.
miR-150-5p	10	10	1.14 (0.41-2.10)	1.20 (0.21-2.13)	0.94	n.s.
miR-142-3p	10	10	0.89 (0.63-2.20)	0.87 (0.61-1.67)	1.02	n.s.
miR-23a-3p	10	10	0.98 (0.72-1.35)	1.03 (0.74-1.31)	0.96	n.s.
let-7e-5p	10	10	0.82 (0.43-3.98)	1.03 (0.51-2.82)	0.79	n.s.
miR-29b-3p	10	10	1.07 (0.50-1.51)	1.08 (0.51-1.33)	0.98	n.s.

miR-374b-5p	10	10	1.19 (0.30-2.70)	0.96 (0.46-2.30)	1.23	n.s.
miR-301a-3p	10	10	0.94 (0.52-1.85)	1.12 (0.46-1.61)	0.83	n.s.
miR-363-3p	10	10	1.00 (0.45-1.70)	1.01 (0.56-1.70)	0.98	n.s.
miR-20a-5p	10	10	1.03 (0.68-1.34)	1.01 (0.59-1.52)	1.01	n.s.
miR-432-5p	9	10	1.18 (0.12-2.34)	1.05 (0.19-2.30)	1.12	n.s.
miR-296-5p	9	9	0.69 (0.10-3.45)	0.99 (0.16-1.55)	0.70	n.s.
miR-145-5p	10	10	0.95 (0.50-2.36)	0.98 (0.69-1.65)	0.97	n.s.
miR-30e-3p	9	9	1.07 (0.43-1.62)	0.80 (0.43-1.75)	1.34	n.s.
miR-374a-5p	10	10	1.11 (0.32-2.86)	1.11 (0.17-2.44)	0.99	n.s.
miR-133a-3p	9	10	0.89 (0.45-2.97)	0.64 (0.13-3.23)	1.37	n.s.
miR-30b-5p	10	10	1.10 (0.49-2.19)	1.03 (0.66-1.89)	1.05	n.s.
miR-188-5p	9	10	0.99 (0.13-3.47)	0.89 (0.21-4.77)	1.10	n.s.
miR-16-5p	10	10	0.99 (0.39-1.76)	1.08 (0.52-1.70)	0.91	n.s.
miR-485-3p	9	10	0.86 (0.12-4.08)	0.94 (0.20-6.10)	0.91	n.s.
miR-136-5p	10	10	1.19 (0.05-3.41)	1.03 (0.38-5.90)	1.15	n.s.
miR-127-3p	10	10	1.44 (0.23-2.97)	1.11 (0.16-2.57)	1.29	n.s.
miR-584-5p	10	10	1.04 (0.27-2.12)	1.07 (0.59-1.76)	0.96	n.s.
miR-221-3p	10	10	1.04 (0.69-1.25)	0.88 (0.73-1.80)	1.18	n.s.
miR-23b-3p	10	10	0.99 (0.78-1.47)	0.95 (0.71-1.47)	1.04	n.s.
miR-532-5p	10	10	1.12 (0.43-2.18)	0.92 (0.52-1.94)	1.22	n.s.
miR-32-5p	10	10	1.16 (0.22-2.14)	1.05 (0.53-1.58)	1.10	n.s.
miR-24-3p	10	10	1.01 (0.83-1.28)	0.95 (0.61-1.57)	1.05	n.s.
miR-574-3p	9	9	0.79 (0.65-1.01)	0.76 (0.40-1.43)	1.03	n.s.
miR-365a-3p	9	9	0.84 (0.44-1.62)	0.87 (0.24-2.98)	0.96	n.s.
miR-140-5p	10	10	1.05 (0.59-1.35)	0.97 (0.73-1.43)	1.07	n.s.
miR-328-3p	9	10	0.84 (0.63-1.38)	0.82 (0.51-1.26)	1.01	n.s.
miR-26a-5p	10	10	0.94 (0.70-1.66)	1.01 (0.75-1.93)	0.93	n.s.
miR-154-5p	10	10	1.21 (0.54-2.81)	0.77 (0.19-3.98)	1.56	n.s.
miR-151a-5p	10	10	0.89 (0.76-1.32)	0.96 (0.70-1.47)	0.93	n.s.
miR-342-3p	10	10	1.01 (0.51-1.75)	1.09 (0.47-1.62)	0.92	n.s.
miR-185-5p	10	10	1.02 (0.55-1.63)	1.10 (0.50-1.49)	0.93	n.s.
miR-126-3p	10	10	1.08 (0.53-1.55)	1.04 (0.69-1.52)	1.03	n.s.
miR-140-3p	10	10	1.01 (0.41-1.72)	1.15 (0.63-1.44)	0.87	n.s.
miR-29c-3p	10	10	1.02 (0.66-1.38)	0.98 (0.64-1.42)	1.04	n.s.
miR-10b-5p	10	10	1.02 (0.52-1.77)	1.08 (0.32-2.14)	0.94	n.s.
miR-874-3p	9	10	0.78 (0.47-1.98)	0.94 (0.48-1.89)	0.83	n.s.
miR-423-3p	10	10	1.06 (0.51-1.54)	0.96 (0.71-1.54)	1.10	n.s.
miR-410-3p	9	10	0.92 (0.29-2.60)	1.04 (0.12-2.78)	0.88	n.s.
miR-126-5p	10	10	1.04 (0.72-1.82)	1.06 (0.21-1.52)	0.98	n.s.

miR-182-5p	9	10	0.94 (0.30-3.07)	0.82 (0.22-3.91)	1.13	n.s.
miR-320a	10	10	0.88 (0.59-1.85)	1.05 (0.59-1.60)	0.84	n.s.
miR-431-5p	9	10	1.14 (0.30-2.26)	0.91 (0.15-2.99)	1.25	n.s.
miR-451a	10	10	1.04 (0.36-1.72)	1.05 (0.46-1.99)	0.98	n.s.
miR-148b-3p	10	10	1.01 (0.54-1.38)	0.99 (0.74-1.25)	1.02	n.s.
miR-744-5p	10	10	1.03 (0.26-1.92)	1.09 (0.66-1.53)	0.94	n.s.
miR-652-3p	10	10	0.99 (0.81-1.22)	0.98 (0.72-1.37)	1.01	n.s.
miR-22-3p	10	9	1.07 (0.79-1.31)	1.05 (0.83-1.34)	1.02	n.s.
miR-34a-5p	10	10	1.02 (0.36-2.99)	1.05 (0.30-4.72)	0.96	n.s.
miR-339-5p	10	10	1.41 (0.21-2.11)	1.02 (0.56-2.57)	1.37	n.s.
miR-93-5p	10	10	1.00 (0.60-1.52)	1.05 (0.53-1.55)	0.96	n.s.
miR-103a-3p	10	10	1.12 (0.59-1.28)	0.96 (0.81-1.24)	1.16	n.s.
let-7d-3p	10	10	1.04 (0.68-1.35)	0.98 (0.85-1.30)	1.05	n.s.
miR-128-3p	10	10	1.02 (0.64-1.59)	1.12 (0.48-1.28)	0.90	n.s.
miR-421	9	10	0.82 (0.73-1.25)	0.80 (0.61-1.29)	1.02	n.s.
miR-329-3p	10	10	0.94 (0.25-2.67)	0.76 (0.31-4.22)	1.23	n.s.

N_{AD} number of Alzheimer patients in which the miRNA was detected, $N_{control}$ number of control patients in which the miRNA was detected, M_{AD} the median of relative expression levels among Alzheimer patients, $M_{control}$ median of relative expression levels among control patients, *FC* fold change of median values, *p* p-values of difference calculated by two-sided nonparametric Mann-Whitney test (not corrected for multiple testing), *n.s*. nonsignificant ($p > 0.05$)
