# Supplementary figures and images for: miRNA expression profiles in cerebrospinal fluid and blood of patients with Alzheimer’s disease and other types of dementia – an exploratory study
Source: Transl Neurodegener. 2016 Mar 15;5:6. doi: 10.1186/s40035-016-0053-5 (PMC4791887; doi:10.1186/s40035-016-0053-5)

LOO classification accuracy

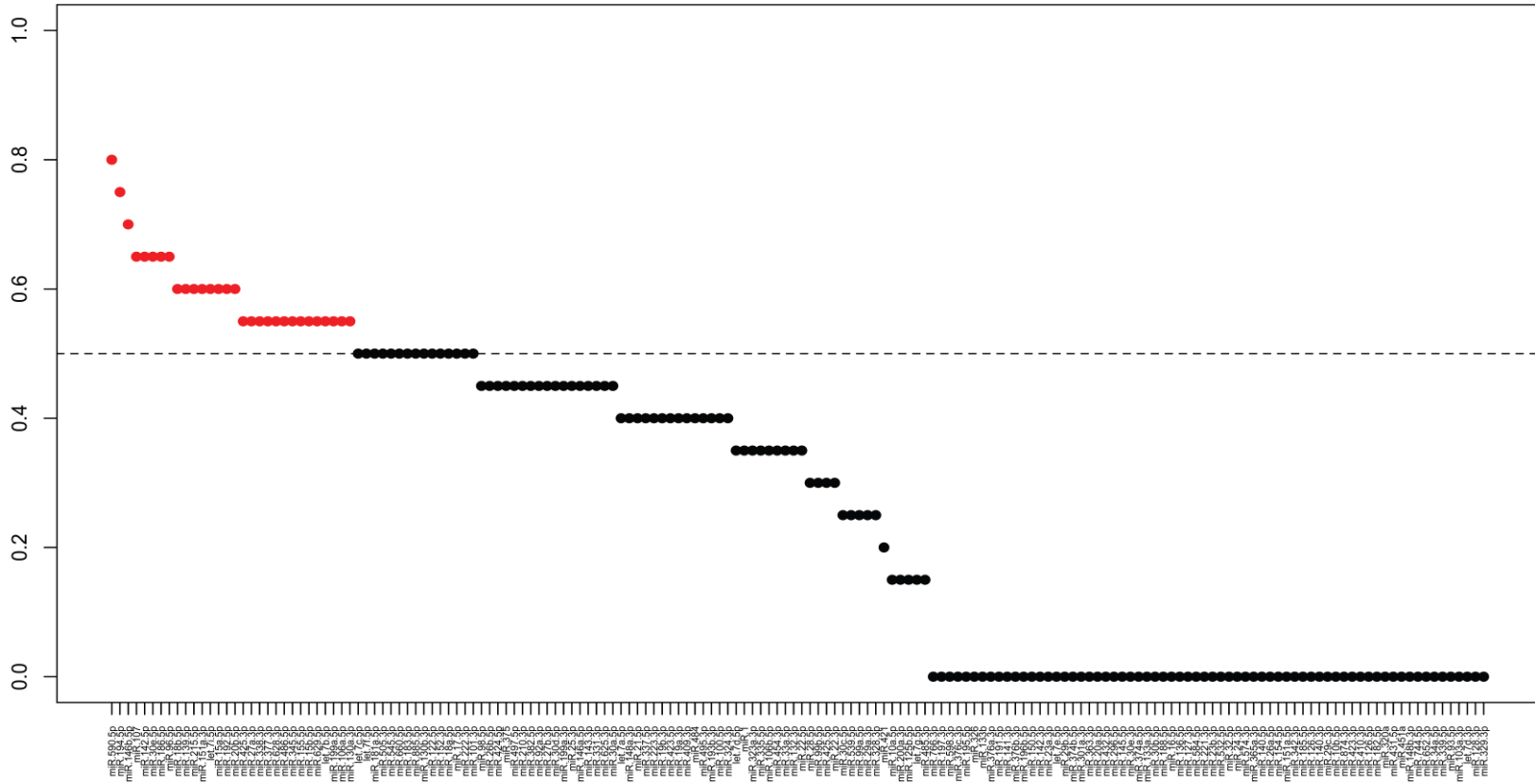

Supplement: Additional file 2: — Logistic regression cross-validated classification accuracies based on a leave-one-out procedure (LOO). The 168 most frequently detected miRNAs in blood are ordered by decreasing magnitude of predictive power. Red color indicates predictors with classification accuracy above random guessing. (PDF 146 kb) [file 40035_2016_53_MOESM2_ESM.pdf]
