# Supplementary material for: miRNA expression profiles in cerebrospinal fluid and blood of patients with Alzheimer’s disease and other types of dementia – an exploratory study
Source: Transl Neurodegener. 2016 Mar 15;5:6. doi: 10.1186/s40035-016-0053-5 (PMC4791887; doi:10.1186/s40035-016-0053-5)
Supplement: Additional file 3: — Estimation of the blood brain barrier dysfunction in patients with dementia based on albumin CSF/serum concentration quotients. AD Alzheimer’s disease, DLB dementia with Lewy bodies, VAD vascular dementia, FTD frontotemporal dementia. Qalb albumin CSF/serum concentration quotient. Qalb limit age dependent upper limit of albumin CSF/serum concentration quotient calculated by the formula ${Q}_{alb}=\left(4+\frac{age}{15}\right)$Qalb=4+age15. BBB blood brain barrier. (PDF 447 kb) [file 40035_2016_53_MOESM3_ESM.pdf]

Additional file 3. Estimation of the blood brain barrier dysfunction in patients with dementia based on albumin CSF/serum concentration quotients

Patient	Diagnosis	Gender	Age	Q_{alb}	$Q_{alb\ limit}$	BBB
1	AD	F	66	8.5	8.4	Leaky
2	AD	M	58	19.1	7.9	Leaky
3	AD	F	56	4.5	7.7	Normal
4	AD	M	59	9.1	7.9	Leaky
5	AD	F	84	8.8	9.6	Normal
6	AD	F	82	6.9	9.5	Normal
7	AD	M	78	13.2	9.2	Leaky
8	AD	F	70	6	8.7	Normal
9	AD	F	72	3.5	8.8	Normal
10	AD	M	75	6	9.0	Normal
11	DLB	M	64	6.7	8.3	Normal
12	VAD	M	79	17.3	9.3	Leaky
13	FTD	F	54	10.8	7.6	Leaky
14	FTD	M	77	9.1	9.1	Normal
15	VAD	F	79	5.9	9.3	Normal
16	FTD	M	79	10	9.3	Leaky
17	DLB	F	68	3.1	8.5	Normal
18	VAD	M	74	7.2	8.9	Normal
19	VAD	M	59	6.2	7.9	Normal
20	FTD	F	61	5.1	8.1	Normal

AD Alzheimer's disease, *DLB* dementia with Lewy bodies, *VAD* vascular dementia, *FTD* frontotemporal dementia. Q_{alb} albumin CSF/serum concentration quotient. $Q_{alb\ limit}$ age dependent upper limit of albumin CSF/serum concentration quotient calculated by the formula $Q_{alb} = (4 + \frac{age}{15})$. *BBB* blood brain barrier
